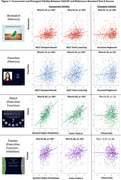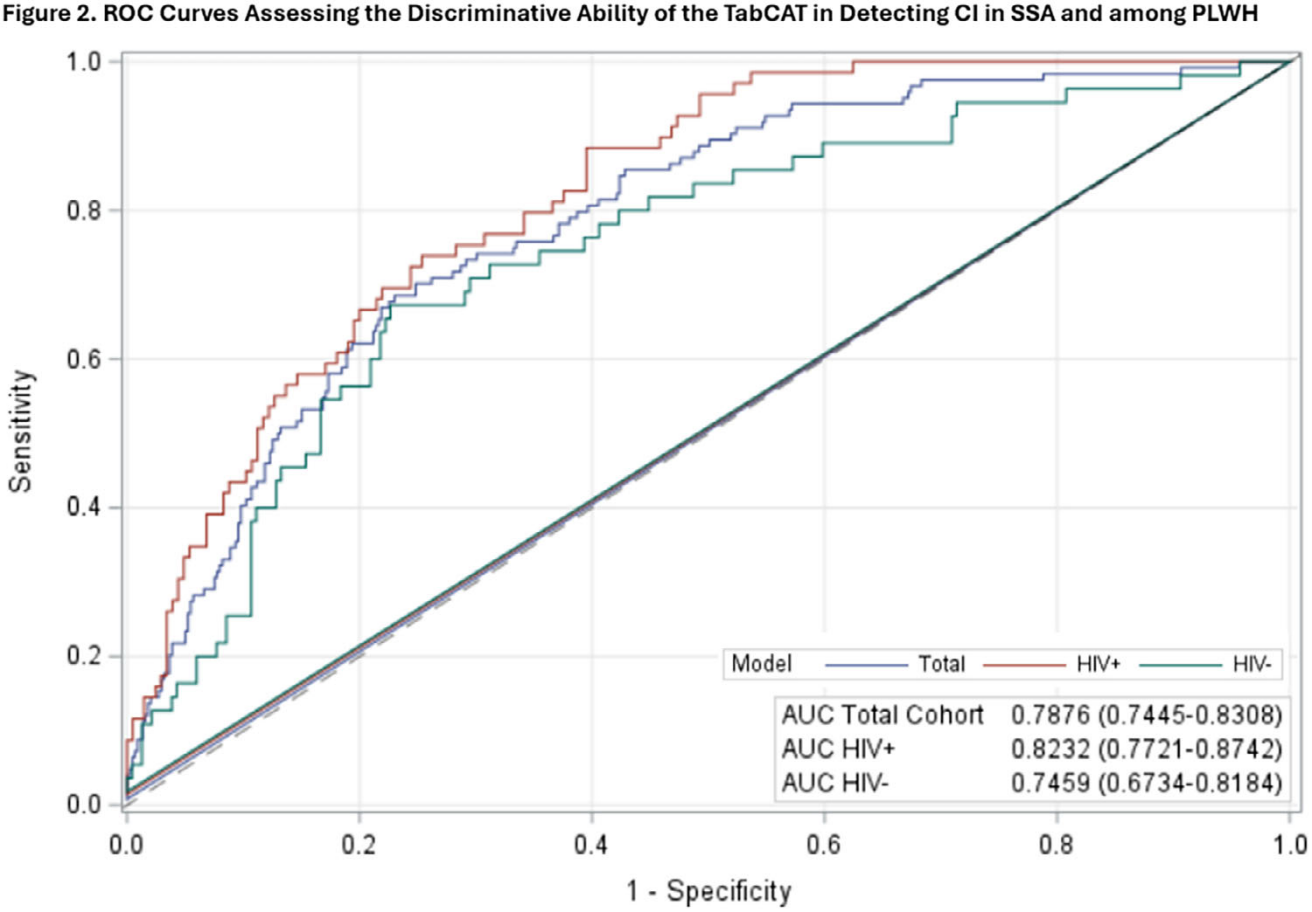# Adaptation and Validation of Brief Tablet‐Based Cognitive Assessment Tool in Uganda

**DOI:** 10.1002/alz70857_102387

**Published:** 2025-12-25

**Authors:** Roslyn Valdespino, Gabrielle Hromas, Chen‐Pin Wang, Robert Paul, Noeline Nakasujja, Zahra Reynolds, Flavia Atwine, Edna Tindimwebwa, Meredith Greene, Eliza Passell, Christine S Ritchie, Susanne S Hoeppner, Alexander C Tsai, Janet Seeley, Amy Werry, Sudha Seshadri, Samson Okello, Stephen Asiimwe, Deanna Saylor, Katherine L. Possin, Elena Tsoy, Mark J Siedner, Jeremy A. Tanner

**Affiliations:** ^1^ University of Texas Health San Antonio, San Antonio, TX, USA; ^2^ University of Missouri, St Louis, MO, USA; ^3^ Makerere University, Kampala, Central, Uganda; ^4^ Massachusetts General Hospital (MGH), Boston, MA, USA; ^5^ Mbarara University of Science & Technology (MUST), Mbarara, Uganda; ^6^ Kabwohe Clinical Research Center (KCRC), Kabwohe, Uganda; ^7^ Indiana University School of Medicine., Indianapolis, IN, USA; ^8^ London School of Hygiene and Tropical Medicine, London, United Kingdom; ^9^ University of North Carolina, Chapel Hill, NC, USA; ^10^ Global Brain Health Institute (GBHI), University of California San Francisco (UCSF); & Trinity College Dublin, San Francisco, CA, USA; ^11^ Memory and Aging Center, University of California San Francisco, San Francisco, CA, USA

## Abstract

**Background:**

Sub‐Saharan Africa (SSA) faces accelerated growth of older adults, older people living with HIV (PLWH), and Alzheimer's Disease (AD). However, there is a shortage of human resources and contextually relevant diagnostic tools to assess cognitive impairment (CI) in the region. The tablet‐based Cognitive Assessment Tool (TabCAT) is a brief digital battery with automated scoring which can be administered by non‐specialists across diverse cultures, languages, and education levels. We assessed its performance in the Uganda Aging Cohort Study (UACS), a prospective cohort study of older PLWH and age‐ and sex‐similar HIV‐uninfected adults in Uganda.

**Method:**

TabCAT tests were translated and culturally adapted through expert review and focus groups. We compared TabCAT scores against performance on a reference‐standard cognitive testing battery previously validated and employed in Uganda. Z‐scores on all tests were derived using a regression‐based normative approach to adjust for age, sex, and education. CI was defined using Jak/Bondi criteria. TabCAT test performances were examined for floor and ceiling effects, with concurrent and divergent validity determined via Spearman rank correlations. Receiver Operating Characteristic (ROC) curves were fit to assess TabCAT composite score discriminative ability for CI in the sample and by HIV serostatus.

**Result:**

TabCAT measures were regarded to have acceptable face and content validity by local experts and focus groups. Participants (*n* = 563, mean age 60±6.5, 50% female, 51% < primary school, 49% PLWH) completed the reference‐standard and TabCAT battery. For TabCAT tests, there were no notable floor or ceiling effects. Correlations between TabCAT and reference‐standard tests were strongest in overlapping cognitive domains (memory, executive function) and weakest in unrelated motor domains (Figure 1). The TabCAT composite score identified CI with good to excellent performance (c‐statistic 0.79; 95% CI 0.74‐0.83), with similar performance in PLWH (Figure 2).

**Conclusion:**

TabCAT tests demonstrate content, concurrent, and criterion validity for identifying CI in a SSA population and in PLWH. Despite variability in concurrent validity on some tests, TabCAT is a promising brief assessment tool to identify CI with generalizability across diverse cultures, languages, education levels, and HIV serostatus. Future studies will examine TabCAT validity for assessing and diagnosing AD and related dementias in Uganda and other resource‐limited settings.